# Triggering Weight Management Using Digital Avatars: Prospective Cohort Study

**DOI:** 10.2196/42001

**Published:** 2023-05-29

**Authors:** Moyez Jiwa, Tafadzwa Nyanhanda, Michael Dodson

**Affiliations:** 1 School of Medicine, Melbourne Clinical School The University of Notre Dame Australia Werribee Australia; 2 Medical Education Unit, Werribee Mercy Hospital Mercy Health Werribee Australia

**Keywords:** weight management, digital avatar, behavior change, calorie awareness, obesity, health promotion, motivation, processes of change, stages of change, BMI, weight, body dysmorphia, diet, exercise, calorie, tool, weight management, digital

## Abstract

**Background:**

There is evidence that showing motivated people with a less-than-ideal BMI (>25 kg/m^2^) digital and personalized images of their future selves with reduced body weight will likely trigger them to achieve that new body weight.

**Objective:**

The purpose of this study is to assess whether digital avatars can trigger weight management action and identify some of the measurable factors that distinguish those who may be triggered.

**Methods:**

A prospective cohort study followed participants for 12 weeks through 5 recorded interviews. Participants were screened for suitability for the study using the Cosmetic Procedure Screening Questionnaire as a measure of body dysmorphia. At interview 1, participants were shown 10 images from a “Food-pics” database and invited to estimate their calorie value. The intervention, the FutureMe app, delivered at interview 2, provided each participant an opportunity to see and take away a soft copy of an avatar of themselves as they might appear in the future depending on their calorie consumption and exercise regimen. Participants completed the readiness for change (S-Weight) survey based on Prochaska Stages of Change Model and the processes of change (P-Weight) survey. Any changes in diet, exercise, or weight were self-reported.

**Results:**

A total of 87 participants were recruited, and 42 participants completed the study (48% of recruited participants). Body dysmorphia was a rare but possible risk to participation. The majority (88.5%) of the participants were female and older than 40 years. The average BMI was 34.1 (SD 4.8). Most people wanted to reduce to a BMI of 30 kg/m^2^ or lose on average 10.5 kg within 13 weeks (–0.8 kg per week). Most participants stated that they would achieve these results by limiting their calorie intake to 1500 calories per day and taking the equivalent of 1 hour of bicycling per day. At interview 1, more participants were in the preparation stage of behavior change than in subsequent interviews. By interview 5, most of the participants were at the maintenance stage. Participants who overestimated the recommended number of calories were more likely to be in the contemplation stage (*P*=.03).

**Conclusions:**

Volunteers who participated in the study were mainly women older than 40 years and beyond the contemplation stage of change for weight management, and those who took weight management action were demonstrated to have a more accurate idea of the calorie content of different foods. Most participants set ambitious targets for weight loss, but few, if any, achieve these goals. However, most people who completed this study were actively taking action to manage their weight.

**Trial Registration:**

Australian New Zealand Clinical Trials Registry ACTRN12619001481167; https://www.anzctr.org.au/Trial/Registration/TrialReview.aspx?id=378055&isReview=true

## Introduction

Having a BMI over 25 kg/m^2^ is recognized as one of the major risk factors for chronic and life-limiting illnesses [1]. Having overweight or obesity is associated with the consumption of more calories than the body requires for physiological functioning. As a consequence, the body stores the excess calories in the form of adipose tissue. This has a negative effect on health and impacts the perception of a person’s physical appearance [2]. Dissatisfaction with physical appearance is one of the most potent drivers for weight management efforts [3]. Among the factors that stimulate an individual to make different lifestyle choices is the desire to achieve a different body shape [4]. There are many diet and exercise programs that an individual can select to achieve that outcome. Individuals will find the means to achieve their goals if they are motivated, feel able to achieve the results, and are triggered to change [5].

This study builds on prior evidence that motivated people with a BMI of >25 kg/m^2^ who are are shown digital and personalized images of themselves with a lower body weight may take steps to achieve that new body shape [6]. The intervention in the previous study did not incorporate a tailored program to achieve the desired results [6]. Rather, participants found their own means to achieve their goals or, alternatively, revisited their goals. This study included participants recruited by a hospital employer. There is evidence that a health services employer is well-placed to promote healthy lifestyles [7].

This prospective cohort study involved convenience sampling of participants who self-reported that they wished to address weight management (BMI>25 kg/m^2^), obese (BMI>30 kg/m^2^), or wanted to maintain their current weight. This study also screened and excluded participants with body dysmorphia. Validated and reliable measures were adopted to follow up on participants’ stages of and processes of change. Any actions taken to achieve a lower BMI were self-reported.

## Methods

### Ethics Approval

The trial was approved by the Mercy Health Human Research Ethics Committee (HR 2019-016) and registered with the Australian New Zealand Clinical Trials Registry (ANZCTR): ACTRN12619001481167. The study adheres to the principles of the Helsinki Declaration. Written informed consent was obtained at inclusion.

### Participants and Recruitment

A convenience sample of participants was recruited from hospital staff, a leisure center, and a clinical trial participant registry. Participants were recruited over 13 months, from November 2020 to December 2021. A total of 634 individuals applied to participate and were screened using eligibility criteria, resulting in 87 final participants. Eligibility criteria required participants to be at least 16 years old, have access to a smartphone, be able to give informed consent, and be interested in or consider weight management (regardless of their current BMI). Individuals with body dysmorphia (a mental illness characterized by constant worrying over a perceived or slight defect in one’s appearance) and those who were pregnant or breastfeeding were not eligible to participate. Hospital staff participants were recruited through the hospital’s website, emails, and flyers.

### Study Design

#### Overview

**Figure 1:**
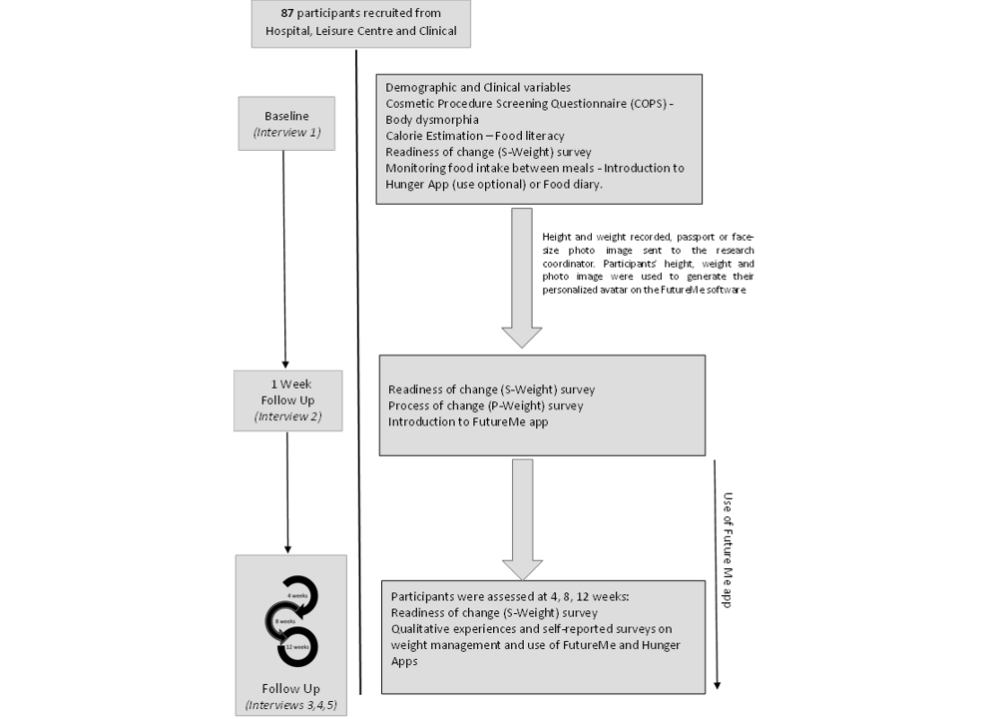
Study flowchart.

The study team followed up with participants for 12 weeks and included telephone and video-recorded interviews. As we conducted the study during the COVID-19 pandemic and in different states in Australia, it was not possible to see participants in person. Validated tools were deployed to collect data. The intervention delivered at interview 2 was a previously tested digital tool to trigger efforts at weight management [6]. The study flowchart is shown in Figure 1.

#### Interview 1

Participants were screened for suitability for the study using the Cosmetic Procedure Screening Questionnaire as a measure of body dysmorphia [8]. Potential participants with suspected body dysmorphia were not eligible to participate and were advised to consult their general medical practitioner for other weight management options.

As a proxy measure of food literacy, participants were shown pictures of up to 10 common foods and asked to estimate their calorie value [9]. Participants completed the 6-item readiness for change (S-Weight) survey, which is based on Prochaska Stages of Change Model [10]. Participants were also invited to monitor their food consumption using an electronic or paper-based food diary. A freely available food diary app was suggested as an option. We did not collect data on the use of any food diaries.

**Figure 2:**
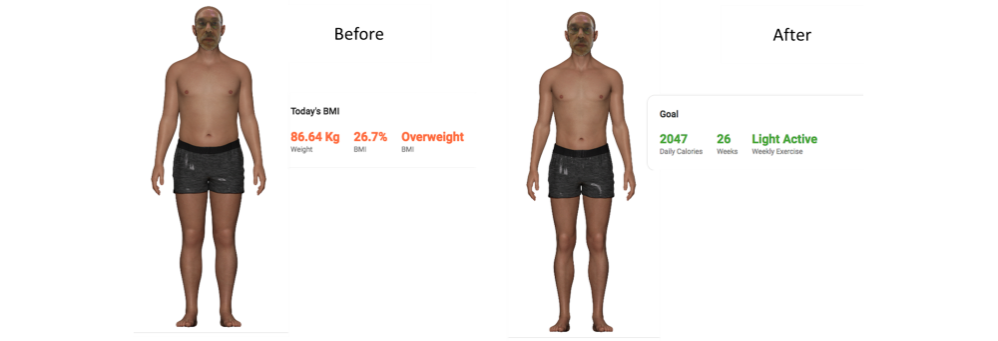
Personalized avatar depicting current weight (before) and goal weight (after) images.

Each participant was invited to attend a review and a second interview 1 week later. Prior to interview 2, participants were reminded to record their height and weight and to send a passport-size or face-size photo image to the research coordinator. A participant’s height, weight, and photo image were used to generate their personalized avatar on the FutureMe software (Archetype Health and Continuum Digital). An example of the avatars and the factors that determined the changes in the before and after images and avatars are shown in Figure 2.

#### Interview 2

Interview 2 was conducted and video-recorded via Zoom. At the interview, participants discussed their food diary with the researcher and undertook the readiness for change (S-Weight) survey. Participants indicated whether they wanted to modify their diet or carry out more exercise (Motivation) and whether they had a strategy to address their food consumption and build more exercise into their routines (Ability). They were then invited to complete a validated process of change questionnaire (P-Weight) about weight management [11]. Following completion of this questionnaire, those who wished to modify their diet or carry out more exercise were referred to freely available resources to assist their efforts and were advised to see their general practitioner or primary care physician or seek a referral for further advice if necessary. All participants were offered web-based resources as well as a screenshot of their “before and after avatars,” from the FutureMe app. The participants chose an avatar that reflected how they would like to look at a time of their choosing in the future (12 or 26 weeks), how many daily calories they would consume, and how many weekly calories they would expend in exercise to achieve that goal. The participants were followed up again 2 weeks later.

#### Interviews 3, 4, and 5

Interviews were conducted at weeks 4, 8, and 12 and video-recorded via Zoom (Zoom Video Communications). Participants repeated the readiness for change (S-Weight) 6-item survey. They were also asked about their experience following the FutureMe app’s recommendations with respect to calorie consumption and exercise. The researcher also recorded any steps they had taken to achieve the goals they set for themselves.

### Instruments

Participants completed several self-report surveys 3 times in the study (weeks 4, 8, and 12), including sociodemographic characteristics and the English versions of the validated P-Weight and S-Weight surveys [11,12].

- Sociodemographic data: information was gathered about age, sex, and BMI.
- Stages of change for weight management (S-Weight): S-Weight is a questionnaire that consists of 5 mutually exclusive items that aim to allocate participants to one of the 5 stages of change for weight management proposed by the transtheoretical model (precontemplation, contemplation, preparation, action, and maintenance) [10].
- Process of change for weight management (P-Weight): the P-Weight questionnaire aimed to determine the processes (attitudes and behaviors to control weight) involved in the change and included 33 items within 4 change processes: emotional reevaluation, weight management actions (WMAs), supporting relationships, and weight consequences evaluation [11]. The participants answered questions on a 5-point Likert scale ranging from 1 (strong disagreement) to 5 (strong agreement). All scores were obtained and then used to calculate and identify the individuals’ process of changing status.
- Calorie estimation: at interview 1, as a proxy measure of food literacy, participants were shown 10 images of common foods obtained from the widely used and validated “Food-pics” database and asked to estimate their calorie value [9]. Participants were shown a visual image of a common snack food and asked to estimate the number of calories in the food by choosing the best answer from multiple options. Participants were also shown 2 images of a man and a woman and asked to estimate the number of calories required for them to maintain their weight, choosing the best answer from multiple options.
- FutureMe app: the FutureMe app was used to provide each participant with the opportunity to see an avatar of themselves as they might appear in the future, depending on their diet and exercise. The participant chose their physical appearance in the future as demonstrated in a full-body avatar, which included their own face and skin color, how many daily calories they should consume, and how many weekly calories they must expend in exercise to achieve that goal at various possible dates in the future, from 12, 26, and 52 weeks. The choice of their “best” look in the future was entirely at the discretion of the participant.

### Statistical Analysis

For sample size calculation, we estimated that the proportion of participants likely to be triggered by the Future Me app to make weight loss attempts from our previous RCT would be 20% [6]. Therefore, we aimed to recruit 100 participants to detect a similar proportion triggered within a 5% margin of error at the 99% confidence level. The estimated size of the sample pool of those eligible to participate was 3951 potential participants (n=600 [Hospital]+1800 [Leisure Centre]+1551 [Clinical Trials Registry]).

All surveys were web-based and developed using the Qualtrics web-based platform (Provo) [13]. The surveys were exported from Qualtrics to Excel (Microsoft Corp). Statistical analyses were performed using SPSS Statistics for Windows (version 27.0; IBM Corp). Descriptive statistics (frequencies and percentages for categorical variables, means, and SDs for variables measured on a continuous scale) were used to summarize the participants’ demographic data, stages of change at each interview, processes of change at interview 2, and survey responses.

For the determination of the stages of change across all interviews, for dropouts or withdrawals, a “Last Observation Carried Forward” strategy was used to estimate all missing measurements [6], whereby each missing value was replaced by the stage of change at the previous interview.

After completion of the P-Weight questionnaire, a raw process score was generated by tallying up the responses to questions. The raw score (with a range of 5-54) was then converted to a 100-point scale to allow for comparisons across processes. We also categorized participants in the action and maintenance stages of change as being in an active phase of change and those in the precontemplation, contemplation, and preparation stages as being in a nonactive phase of change. We described mean scores with SDs for participants with active and nonactive phases of change and compared means using a 2-tailed independent *t* test with a significance level set at *P*=.05. The stages of change (S-Weight) data for the participants were recorded as categorical data and compared to P-Weight scores (numeric data). Comparisons between groups were performed using ANOVA and the Student *t* test for continuous variables and the chi-square test for categorical variables. The significance of the results was considered with *P*< .05.

For calorie estimation, responses were grouped as follows: underestimation (any response selected with a caloric value lower than the accurate value), accurate estimation (correct estimation of calories), and overestimation (any response selected with a caloric value higher than the accurate value foods). Foods with more than 150 calories were categorized as high-calorie foods and those below 150 as low-calorie foods. Descriptive statistics were conducted, and the chi-square test was used for association tests. Open-ended survey responses from participants’ experiences of using the FutureMe app and weight management were manually coded inductively by emerging themes.

## Results

### Overview

At interview 1, two potential recruits to the study were found to not be eligible on the basis of potential body dysmorphia. A total of 87 participants were recruited, and 42 participants completed the study (48% of recruited participants). Demographic data are presented in Table 1. The majority of participants were female, and all but 2 participants were over 25 years. The average BMI was 34.1 (SD 4.8). Most people wanted to reduce to a BMI of 30 or lose on average 10.5 kg within 13 weeks (–0.8 kg per week). Most participants stated that they would achieve these results by taking a moderate amount of exercise, equivalent to using 580 calories per day or 1 hour of cycling per day, and eating no more than 1500 calories per day on average.

**Table 1:** Study participants’ demographics.

		Interview 1 (N=67)	Interview 2 (N=75)	Interview 3 (N=63)	Interview 4 (N=47)	Interview 5 (N=42)
**Gender, n (%)**						
	Male	9 (10.3)	9 (12)	8 (12.7)	8 (17)	8 (19.1)
	Female	77 (88.5)	66 (88)	55 (87.3)	39 (83)	34 (80.9)
	Nonbinary	1 (1.2)	N/A^a^	N/A	N/A	N/A
**Age (years), n (%)**						
	18-25	2 (2.3)	N/A	N/A	N/A	N/A
	26-40	21 (24.1)	19 (25.3)	15 (23.8)	11 (23.4)	10 (23.8)
	41-50	19 (21.8)	14 (18.7)	12 (19.1)	10 (21.3)	9 (21.4)
	51-60	30 (34.5)	28 (37.3)	24 (38.1)	15 (31.9)	13 (31)
	<60	15 (17.3)	14 (18.7)	12 (19)	11 (23.4)	10 (23.8)

^a^N/A: not applicable.

### Estimation of Daily Caloric Requirements

At interview 2, one in 3 participants accurately determined the recommended number of calories required by men and women. A significant percentage of participants (32.3%) overestimated the requirement, while 26.4% and 8% of them underestimated and did not know, respectively.

### Calorie Estimation of Foods

Overall, more participants tended to overestimate the calorie content of low-calorie foods compared to high-calorie foods. A higher percentage accurately determined the number of calories in high-calorie foods compared to low-calorie foods. Participants older than 40 years were more likely to overestimate the calorie content of calorie-dense foods, whereas participants aged between 26 and 40 years were more likely to give an accurate estimation (*P*=.05). Participants who overestimated the recommended number of calories were more likely to be in the contemplation stage (*P*=.03). There were no significant associations between calorie estimation and stages of behavior change.

### Stages of Change

A higher number of participants at interviews 3 and 4 were in the action stage compared to earlier interviews, and there were fewer participants at interview 5 in the preparation stage compared to interview 1 (*P*=.03). By interview 5, most of the participants were in the action and maintenance stages.

### Processes of Change

At interview 2, emotional reevaluation was the most common change process used.

We categorized participants in the action and maintenance stages of change as being in an active phase of change and those in the precontemplation, contemplation, and preparation stages as being in a nonactive phase of change. The mean processes of change scores with SD are provided in Table 2 below.

Participants in the active phase of change (n=59) had significantly higher mean WMA scores (active phase mean WMA score 53.8, SD 11.9 vs nonactive phase mean WMA score 45.6, SD 16, *P*=.02) than those in the nonactive phase of change (n=16).

**Table 2:** Active and nonactive phases—processes of change mean scores.

	Emotional reevaluation (EmR)	Weight consequences evaluation (WCE)	Supporting relationships (SRs)	Weight management actions (WMAs)
Nonactive phase, mean (SD; n)	75.3 (10.9; 16)	59.6 (18.4; 16)	58.4 (16.4; 16)	45.6 (12.3; 16)
Active phase, mean (SD; n)	76.8 (9.7; 59)	54.3 (14.5; 59)	57.5 (15.2; 59)	53.8 (11.9; 59)
Total, mean (SD; n)	76.5 (9.9; 75)	55.4 (15.4; 75)	57.7 (15.4; 75)	52.1 (12.4; 75)

### Self-reported Attempts at Weight Management

Approximately half of the participants adopted positive weight management actions in the 4 weeks prior to interview 5. For those participants who completed interview 5 (21.4%) reported reducing their calorie intake in the 4 weeks prior to the interview, and 14.3% of them had not changed their diet but claimed to be exercising regularly.

More than half the participants (54.8%) reported following the FutureMe recommendations at least 4 times a week or more. Many participants (45%) self-reported that it was difficult to limit their calorie intake or to exercise more.

Despite some participants finding it difficult to consistently eat less and exercise more, others described experiencing positive results. For example, a few reported losing weight and changing their exercise and eating habits. At the end of the study, 3 male participants and 3 female participants who chose to reply to the open-ended questions reported positive results. Some described how participating in the research and using the FutureMe app held them accountable and motivated them (Textbox 1).

**Participants’ description of participating in the research.**

- “Lost 3kg so far” [Participant 75, 41-50 years, Male]
- “Lost 11.3kg since starting, changing my target to 95, rather than 100 kg initial goal” [Participant 78, >60 years, Male]
- “Hit goal weight from sticking to calorie goal, now trying to maintain” [Participant 24, >60 years, Female]
- “It has been fairly easily once gotten used to reduced calories and calorie counting in diary keeps you on track” [Participant 59, >60 years, Female]
- “General well-being has improved and managed to lose weight from following recommendations and exercising more” [Participant 21, 41-50 years, Male]

## Discussion

### Principal Results

Upon deploying FutureMe, a similar proportion of the participants as reported previously may have been triggered to take weight management actions [6]. Shifts in the movement of participants between the different stages of behavioral change from interviews 1 to 5 were evident. At interview 1, more participants were in the preparation stage. By interview 5, most of the participants were at the maintenance stage. In this active stage of change, the data suggest that participants were more likely to take weight management action. Therefore, it is plausible and consistent with the self-reported fact that most participants who completed the study were actively working to manage their weight.

It was also evident from the data, as well as from the literature, that most of the participant’s knowledge about the calorie content of food was not accurate [14]. The data suggest that those with a more accurate understanding of the calorie content of food were more likely to be triggered to take weight management action. We also acknowledge that our participants’ ability to take the necessary action may have been limited by other factors that were not explored in this observational study but have been the focus of study by others [15].

The data add to the literature that motivated people can be triggered to make attempts to enhance their lifestyle by personalized avatars of their future selves [16]. Most of the data so far relate to virtual reality images and not to the types of avatars used in this study. The targets for weight loss set by the participants in this study were challenging. A weight loss of 10 kg in 13 weeks starting with a BMI of 34.1, well into the obese range, would require major lifestyle changes. Participants were invited to reconsider these targets at interview 2. At that interview, they were shown how they would have to restrict their diet and increase their exercise to achieve the desired appearance. Knowledge of the significant cost of diet restriction and commitment to regular exercise, as suggested by FutureMe, did not change participants’ weight loss goals. Although these goals may be ambitious, there is some evidence in the literature that higher goals motivate weight loss more than undermine effort [17].

### Comparison With Prior Work

Prior work using the FutureMe app intervention focused on documented weight loss and the timing of showing subjects their future avatars from the point of recruitment [6]. In this study, we focused on the participants’ knowledge of calorie values in food, the stage of change, and the process of change associated with weight management. This may help health professionals identify those who might be triggered as well as when to introduce the avatars. We also note that in our studies so far, the actual weight loss has been much lower than the target of 0.8 kg per week set by participants. Both the ability to achieve a self-selected target and the initial onboarding with reference to calorie awareness may be important for those who attempt weight management.

Though limited research has been published, the inclusion of avatar technology in weight loss interventions triggers weight maintenance [6,18-20]. Avatar personalization, with the person’s actual face, skin color, hairstyle, and personal choices of diet and exercise, seems to be important in triggering weight management [6,21].

### Limitations

One limitation of our study is that we did not achieve the target of 100 participants. Most volunteer participants were older females; this may reflect the appeal of the intervention to that demographic, but we cannot report on its value in other groups. The study was also conducted at a time of pandemic-related lockdown across state borders in Australia, so weight loss was not confirmed by in-person weight measurements. Therefore, we were unable to verify any weight loss reported. We were also advised to adopt self-reports, as those who might fail might be distressed if they were to reflect on these data. We, therefore, acknowledge that social desirability bias cannot be ruled out. There was also a high attrition rate in the study. However, we had no indication that those who dropped out of the study had specific characteristics. Additionally, the observed attrition rate of 52% is on par with most weight-loss trials, where attrition is commonly at least between 20% and 50% [22].

### Conclusions

In this study, participants in the FutureMe intervention mainly included women older than 40 years. The most promising results were for those who could more accurately estimate the calorie content of food and were beyond the contemplation stage of change. The participants were offered a trigger to manage their weight, primarily in the form of an avatar depicting their future selves after a period of calorie restriction and increased exercise. As in the previous trial, this may have been effective for many participants [6]. Most of those who were followed up at 12 weeks reported actively working to manage their weight.

However, most participants set very ambitious goals for calorie restriction and exercise regimens that could not be achieved in the time they had nominated to achieve those results. It appears that most people who opted to participate were unlikely to succeed based on their early stage of change, their inaccurate estimates of the calorie content of food, and the ambitious goals they set for calorie restriction and exercise regimens. Motivation and being triggered are not sufficient to achieve behavior change if the person is not able to achieve their goals. Our data suggest that some aspects of knowledge that are necessary in regard to weight management were lacking in the volunteers in this study.

## Abbreviations

WMA: weight management action
